# How Do Young Women Perceive Adult Responses to the Disclosure of Their Self-Harm and What Is the Impact of That Perception?

**DOI:** 10.3390/ijerph22121879

**Published:** 2025-12-17

**Authors:** Demee Rheinberger, Isabel Mahony, Anastasia Hronis, Samantha Tang, Helen Christensen, Fiona Shand, Alexis Whitton, Katherine Boydell, Aimy Slade, Alison L. Calear

**Affiliations:** 1 Black Dog Institute, University of New South Wales, Sydney, NSW 2031, Australia; d.rheinberger@blackdog.org.au (D.R.); samantha.tang@blackdog.org.au (S.T.); h.christensen@blackdog.org.au (H.C.); fionas@unsw.edu.au (F.S.); a.whitton@blackdog.org.au (A.W.); k.boydell@blackdog.org.au (K.B.);; 2 Discipline of Clinical Psychology, Graduate School of Health, University of Technology, Sydney, NSW 2007, Australiaanastasia.hronis@uts.edu.au (A.H.); 3 Faculty of Medicine and Health, University of New South Wales, Sydney, NSW 2031, Australia; 4 Centre for Mental Health Research, The Australian National University, Canberra, ACT 2601, Australia

**Keywords:** self-harm, non-suicidal self-harm, disclosure, response, impacts, young women, adults

## Abstract

Rates of self-harm amongst young women are rising. However, only half of individuals disclose self-harm, and when they do, they may be met with responses that can be harmful or helpful to recovery. The aim of the current study is to understand how young women perceive adult (e.g., parents, health professionals) responses to their self-harm disclosures, and the impact these responses have on them. Semi-structured interviews were conducted with young women (N = 27, M age = 20.9, SD = 2.1) reporting a history of self-harm. Reflexive thematic analysis was used to analyse the data, with three main themes generated: (1) the young woman’s needs were diminished, (2) the confidant’s response was not attuned to their needs, and (3) the confidant’s response was attuned to their needs. The first theme reflects responses that felt dismissive or elicited feelings of discomfort or shame. The second theme captures responses that failed to resonate with the participant’s needs or were unhelpful or invalidating. The third theme represents responses that elicited feelings of being cared for or validated. Future interventions could focus on educating parents and health professionals about the best approaches to responding to self-harm disclosures that promote future disclosure and recovery.

## 1. Introduction

Self-harm occurs when an individual intentionally causes damage or pain to their own body [1]. This behaviour can serve a variety of functions, including managing and regulating emotions, connecting with others, self-punishment, or managing suicidal thoughts [2,3,4,5,6,7]. In Australia, hospitalisations due to self-harm have almost doubled amongst older adolescent females (aged 15–19 years) in the last decade, and it is estimated that self-harm could be almost ten times greater for non-hospitalised self-harm [8]. Rates of self-harm in adolescent males have not increased at the same rate as females [8]. Self-harm is associated with increased risk of suicide [9,10], with one study demonstrating that almost all adolescent participants with a suicide attempt engaged in previous self-harm [10]. Self-harm is also associated with an increase in other risky behaviours [11], poorer outcomes for education and employment, greater likelihood of engaging in substance use, and increased mental health concerns [12,13].

Studies show that over half of university students who have self-harmed had not informed someone about their self-harm [14,15]. There is a well-established body of literature identifying reasons why someone would choose not to disclose self-harm, including perceived stigma, shame, judgement, and misunderstanding [14,16,17], as well as concerns for how the other person may be affected by the disclosure, and reluctance to feel like a burden to others [17]. Furthermore, for those who do disclose experiences of self-harm, there is no evidence that disclosure guarantees further help-seeking or more positive coping strategies. A cross-sectional survey of over 12,000 randomly selected Australians in the community demonstrated that whilst most participants disclosed their self-harm to at least one family member or friend, less than half of the participants sought formal help [18]. Individuals with more severe self-harm or suicidal ideation may also be more likely to disclose their experiences to parents or friends but less likely to seek help from formal sources [19], which is particularly concerning given the uncertain effectiveness of informal sources of support.

Research has shown that confidant responses to self-harm disclosure can vary, partly reflecting the intense emotions that can surround a disclosure [20,21], as well as feelings of being under prepared or uncertainty about how to respond [21]. As such, individuals disclosing their experience of self-harm may be met with negative reactions and judgement from confidantes, following self-harm disclosure [22]. Such responses can involve silence, inaction or avoidance of the topic [17], or highly emotional reactions such as anger, tearfulness, dismissiveness, trivialisation of the self-harm, and gossiping amongst peers [5]. More positively perceived responses have included emotional support and sympathy [23], understanding and acceptance [17], and ongoing support [5].

The impact of responses to self-harm disclosure depends on the response itself and how the response is perceived by the individual disclosing self-harm. Negative disclosure experiences may detrimentally impact an individual’s choice to disclose again, delay future disclosures due to increased hesitation and caution [21], or potentially trigger subsequent self-harm episodes [24]. Conversely, if the confidant responds with acceptance, understanding, and empathy, then this can reduce the shame associated with self-harm [17] and encourage future disclosure and help-seeking that may ultimately reduce future risk of suicide [17,21,25,26]. Additionally, emotional support style responses are associated with decreased levels of depression [26]. This is notable for young female adolescents, as higher levels of depressive symptoms at a given time are associated with higher levels of self-harm over time and vice versa [27].

Interestingly, the impact of a response to a disclosure may not be dependent only on whether the experience was classified as positive or negative but also the specific elements within the response. A study by Ammerman and McCloskey [23] found that willingness to disclose again was dependent on the specific style of “positive” responses, specifically emotionally supportive responses (e.g., listened to the discloser’s thoughts and feelings, showed understanding of their experiences, or did not make judgements) vs. tangible aid style responses (e.g., encouraging the individual to seek counselling, providing distraction, or offering to help them seek medical attention). Whilst both response styles were perceived as helpful by participants, they found that higher levels of emotional support increased willingness to disclose, whereas tangible aid style responses were not associated with someone’s willingness to disclose again.

Importantly, little research has examined the effect of disclosure as a function of the type of confidant (adult or peer). The type of confidant may influence recovery differently. Hasking, Rees, Martin, and Quigley [25] found that the severity of self-harm reduced only when the youth reported disclosure to an adult as opposed to a peer. They also found that the likelihood of seeking help for an emotional and/or behavioural problem (not specifically for self-harm) was increased only in cases of disclosure to a peer as opposed to an adult [25].

Understanding the impact of different types of confidantes is crucial for encouraging help-seeking and developing interventions to support recovery from self-harm. Friends can provide first-line support for adolescents who self-harm, they can be both physically and emotionally available when supporting the adolescent to cease self-harming and when disclosing to adults [28]. Adults, such as parents, teachers, crisis support workers, and health professionals (e.g., GPs, psychologists, and hospital staff) can play a different role than peers in a young person’s recovery. Parents can provide ongoing support and may be more heavily involved in the young person’s day-to-day recovery [5,29], while other adults, such as health professionals and crisis support workers, may play a more formal role in the person’s treatment. The role of teachers can be mixed, with some teachers providing emotional support, while others may act as an intermediary between the young person, their parents, and services.

Understanding the specific impact of adult responses to self-harm disclosures by young women is crucial for supporting help-seeking behaviours, providing ongoing emotional support, and developing appropriate interventions. Importantly, several studies highlight the need for additional training and support for parents, teachers, and professionals in relation to responding to and managing self-harm [29,30,31,32]. The aim of the current study is to understand how young women with current or past self-harm perceived adult responses to disclosure of self-harm, and the subsequent impact of these responses. In the current study, adult confidantes include parents, teachers, crisis support workers, and health professionals (e.g., GPs, psychologists, and hospital staff).

## 2. Materials and Method

### 2.1. Ethical Considerations

This study received ethics approval from the Human Research Ethics Committee (HREC) at the University of New South Wales HC230437. The current study is a part of a larger study investigating self-harm experiences in young women in Australia (e.g., [33]).

### 2.2. Participant Recruitment

Participants were recruited through advertisements on Black Dog Institute’s (BDI) website and social media accounts (Facebook, Instagram, and LinkedIn) between October and November 2023. Advertisements directed potential participants to a website with information about the study. Participants were deemed eligible if they (i) endorsed a history of or current deliberate self-harm (either with or without suicidal intent), (ii) were between 16 and 24 years of age, (iii) identified as female, (iv) could speak and understand English clearly, and (v) were living in Australia. As part of the screening process, to ensure acceptable understanding of study requirements, participants aged 16–17 years were required to complete the Gillick competency assessment [34]. While attempts to recruit participants in this age group were made, no 16–17-year-olds volunteered to participate in the research. To ensure eligibility criteria was met, all participants who provided informed consent were directed to complete a 2 min screening survey administered via Qualtrics. Ineligible participants were directed to a page with information on helplines. Eligible participants were assigned to a researcher who contacted the participant to organise voluntary participation in an interview via Zoom. Participants were provided the opportunity to speak with a registered clinical psychologist to discuss any concerns or distress that arose during the interview. Participants were re-imbursed for their time with a gift-card. Sample size was determined based on the notion of information power [35], which proposes that a moderate–large sample of approximately 25–30 young women was necessary to ensure a fine-grained exploration of individual experiences given the broad aim of the interviews examining a specific experience (self-harm).

### 2.3. Data Collection

Data was collected through semi-structured one-on-one interviews. Interviews lasted between 41 and 106 min (mean = 70 min). Interviews were conducted online via Zoom, using an interview guide that was collaboratively created with researchers and individuals with lived experience of self-harm. The interview guide included questions on self-harm history, circumstances/stressors leading to self-harm behaviour, impulsivity of self-harm episodes, method choice, help-seeking behaviour, and service preferences (see Supplementary S1). Researchers conducting the interviews were Clinical Psychology Masters students and provisional psychologists. All interviews were audio recorded and transcribed using a confidential third-party service and deidentified. No interviews were reviewed or validated by participants.

### 2.4. Analytic Approach

Data analysis followed an inductive reflexive thematic analysis approach [36]. Reflexive thematic analysis was chosen because it allows the researchers to examine and consider how their world views and expertise shape the way that sense is made of the data. First, researchers (IM and DR) engaged in familiarisation with the data by reading and re-reading interview transcripts. Interview transcripts were individually coded using NVivo (version 12) [37] to explore experiences of self-harm disclosures to adults and their subsequent impacts. Adults encompassed any person who was neither a peer nor romantic partner, including parents, caregivers, teachers, and health practitioners (such as psychologists, nurses, doctors, general practitioners (GPs), or crisis support workers). Two researchers (IM and DR) engaged in independent line-by-line coding of the transcripts. At the time of analysis, IM was a clinical psychology master’s student with clinical experience in adult populations and DR was a PhD candidate with extensive experience conducting qualitative research in high-risk populations. Both IM and DR believe in the humanistic approach to healthcare and believe that all individuals should be treated which respect, empathy, and kindness in all interactions. Both researchers recognise that the way in which people respond to disclosures of self-harm, suicide, or psychological distress can significantly impact further engagement with services. Codes were generated over several iterative stages, to ensure experiences of self-harm disclosure were comprehensively understood. Throughout this process IM and DR discussed codes in-depth, compared discrepancies, and engaged in open reflection by questioning how their assumptions informed the interpretation of the data. Once agreement had been reached, the researchers grouped related codes to generate themes. Initially, themes were developed independently, and then the two researchers discussed each proposed theme structure to ensure all perspectives were considered. The final set of themes were reviewed between the two researchers and a third researcher (AH) who was not involved in the original coding process. The final set of themes were generated and agreed upon by the three researchers. IM and DR engaged in reflection throughout the analysis process through journaling and discussions to explore their positionality and the influence of their biases and world view on the analysis. Rigour was maintained through in-depth engagement with the data, a group approach to analysis, maintenance of a detailed audit log, and reflexive notetaking.

## 3. Results

### 3.1. Participants

Twenty-seven young women participated in interviews to explore personal experiences of self-harm. The mean age of participants was 20.9 years (SD = 2.1), ranging from 18- to 24-years. A total of 77.8% of participants reported being employed part-time, while 22.2% were unemployed. Two-thirds of the participants were currently studying, with 37% studying full-time and 29.6% studying part-time. All participants endorsed receiving a diagnosis of at least one mental health condition and none identified as Aboriginal or Torres Strait Islander. The most common mental health diagnoses reported across participants were depressive disorders (77.8%), anxiety (63%), and feeding/eating disorders (25.9%). Other disorders included neurodevelopmental (25.9%), obsessive compulsive (14.8%), and trauma-related (14.8%).

### 3.2. Thematic Analysis

Thematic analysis generated three themes exploring young women’s perception of responses to disclosures of self-harm: (1) the young woman’s needs were diminished, (2) the confidant’s response was not attuned to the needs of the young woman, and (3) the confidant’s response was attuned to the needs of the young woman (see Table 1). Distinction in adult type (e.g., parent, healthcare professional, etc.) is noted within each code where appropriate; no distinction is provided when responses were not specific to a specific adult type.

### 3.3. The Young Woman’s Needs Were Diminished Through the Response

Participants described negative and, in some cases, harmful experiences where the adult responded in a way which diminished or lessened the young woman’s needs. The codes within this theme included responses that dismissed the self-harm or elicited feelings of discomfort or shame.

#### 3.3.1. Self-Harm Was Dismissed

More than half the participants described instances where they felt their self-harm experiences were dismissed by an adult. This included hospital staff, healthcare professionals, or parents responding in a way that felt dismissive, minimising, or ignored the self-harm. Mental health professionals tended to prioritise coping strategies as opposed to the underlying reasons for the self-harm behaviour. Parents typically responded by not intervening or not discussing it with the young woman or making jokes about the young woman’s experience or becoming visibly angry. In response to feeling dismissed, some participants reported engaging in more severe self-harming behaviour or attempting suicide.

*“*[My parents] *saw it one day, but no intervention came of that, so it just wasn’t spoken about again … which just showed me that it was something to hide and be shameful of and not something that needed any intervention.”*

(Participant 20)

*“And she took me to the hospital and then they basically said that there was nothing that they could do for further treatment. And then later in that week I attempted suicide. … getting told that you are basically not extreme enough, that does result in more of an environment where it’s like, I am valid because I’ve hurt myself enough.”*

(Participant 17)

#### 3.3.2. Young Women Felt Uncomfortable

Almost a third of participants described instances where the adult’s response caused them to feel a sense of discomfort or uneasiness. Typically, this occurred in the case of health professionals, for example, young women being asked to remove their clothes during a GP consultation. Young women reported that these interactions made them feel othered or alienated within the healthcare system. Participants also discussed feeling uncomfortable when parents repeatedly asked about fresh self-harm injuries or when they had a bigger emotional response than participants felt was warranted. This made the young women reluctant to discuss their self-harm with parents or encouraged them to hide their injuries.

*“Along with everything else, I found it really hard to explain why, and sometimes being asked why, it’s just kind of, how do you explain that at that time? And that just definitely made me feel uncomfortable.”*

(Participant 16)

The subsequent impact for some participants was that they became more hesitant to engage in further help-seeking, disclose further detail of their self-harming behaviour or disclose it at all in the future.

*“I think what makes a difference is if people are kind of scared to talk about it, then you just feel uneasy about it. I had a psychologist … and you could see that she was uncomfortable and I was like, oh, I don’t want to talk about it …. it makes you feel like it’s a really bad thing that you can’t even talk about it with clinicians.”*

(Participant 13)

#### 3.3.3. Young Women Felt Ashamed

Some participants narrated experiences where an adult’s response resulted in them feeling guilty or ashamed of the self-harm. The majority of these participants described this experience in the context of how their parents responded, which included responses such as looking visibly upset, asking a lot of questions in an interrogative manner, or encouraging the youth to cover up their self-harm.

*“I saw the expression on his* [father] *face and that was something that was really painful … particularly when I’d become aware of how much it was hurting people around me, guilt when I was really working on trying not to do it.”*

(Participant 1)

*“… my parents would be like, what are you doing? You’re bringing shame to the family. What is this? What are you doing? This is unreasonable. … now I’m sad and I feel embarrassed, and I have shame.”*

(Participant 5)

These responses left the young women feeling as though they were a bad person for self-harming or contributed to hesitation to seek help in the future.

*“… when you’re just sort of interrogated, you’re very much given the feeling that … it’s a bad thing, or I’m a bad person for having done that. And it kind of shuts off any communication about it because you feel like you’re just going to get in trouble if you tell them.”*

(Participant 3)

In terms of health professionals, one young woman described a GP who attempted to use religious messaging to motivate her to cease the behaviour.

### 3.4. The Confidant’s Response Was Not Attuned to the Needs of the Young Woman

Participants recounted instances where an adult’s response failed to resonate with the young woman’s needs. The codes within this theme included the young woman’s needs being misunderstood and the adult not providing meaningful help or assistance to them.

#### 3.4.1. Needs Were Misunderstood

Almost a third of participants described experiences where they felt invalidated by an adult’s response to a self-harm disclosure. They described experiences where an adult responded in a way that made them feel as if the adult did not understand their emotional needs or experience. For some participants, parental responses did not meet their emotional needs, where the reaction was either too intense (e.g., responding with anger, questioning the young woman’s reasons for self-harming, or parents ‘playing the victim’ by switching the focus to themselves), or not emotional enough (e.g., responding in a neutral manner as opposed to an emotional one) which led to feeling lost.

*“… I felt sad and a lot of the time I felt pretty emotionless in a way. So I kind of wanted someone to feel like a strong emotion for me. …, I kind of wanted someone to have some sort of reaction that would help me gauge what I was supposed to be feeling or something like that.”*

(Participant 15)

For others, this misunderstanding was in the context of help-seeking and the intervention not meeting their needs at the time. Examples of health professionals’ responses were commenting on scars, focusing on other aspects of the young woman’s presentation, or advising that the young woman’s scars were not deep enough when seeking medical attention. Invalidating responses could then contribute to hesitation to disclose self-harm again in the future or to feeling alienated.

*“I think a lot of the advice I was getting at the time was distract yourself … And I kind of didn’t feel like I had the capacity to … I kind of felt like it was just all missing a massive part of the sort of thing that I was getting from self-harm.”*

(Participant 10)

#### 3.4.2. Response Was Unhelpful

A third of participants discussed experiences where the adult responded in a way that was unhelpful. Participants described feeling as if the adult’s treatment, response, or choice of intervention did not change their circumstances in a meaningful way. In these instances, participants tended to report not experiencing a strong positive or negative emotional response to the confidant’s actions. For most of these experiences, the adult was a health professional (psychologist, doctor, or nurse) or another professional (such as teacher or mental health call centre).

*“*… [Crisis Call Centre] *weren’t good in the moment because I know this can’t really be helped, but just being put on hold was not helpful. And a lot of the time the whole message was, if it’s urgent, go to the emergency department. But I’d done that and so that wasn’t something that I wanted to do again. So that wasn’t helpful.”*

(Participant 4)

While some interventions or responses did not make a meaningful change to their experience, some unhelpful responses could also lead to further self-harm.

*“They’ve just turned into reasons to self-harm. … Like with the ice for example, it was, oh, now I can self-harm because I tried it and it didn’t work.”*

(Participant 24, in response to a clinician suggesting applying ice to the skin as an alternative to self-harm)

### 3.5. The Confidant’s Response Was Attuned to the Needs of the Young Woman

Participants also described instances where the adult’s response was attuned to their needs, which provided a positive and sometimes meaningful experience in their journey. The codes explored within this theme included a sense of feeling cared for and their struggles being acknowledged.

#### 3.5.1. Young Women Felt Cared For

A third of participants spoke about experiences in which an adult responded in a way that made them feel looked after and cared for. Some of these participants described this experience in response to someone offering to help or engaging in a small but meaningful action, like asking what they needed or letting them discuss their situation. For some participants, this was when a parent took their self-harm seriously and took them to seek medical intervention, helping the young person realise their safety is important.

*“They’ve always been really good. They brighten your day up a little bit when you’re feeling down, if they’re just up for a chat and just being nice and it’s always nice to have a doctor who cares. It makes you feel seen and heard a bit more.”*

(Participant 24)

*“I was suicidal, and I told mum that I was feeling that way and that I needed to go to the hospital. … And she took me to the hospital … I’ve also struggled because it’s normalized for me with self-harm. … And so when it’s brought to your attention by someone else … I always found it helpful because I was like, wow, this is something that I should be more aware of in terms of my safety.”*

(Participant 17)

#### 3.5.2. Struggles Were Acknowledged

Almost half of the participants described instances where their experience and needs were understood or validated by the adult acknowledging their self-harm and distress. Typically, this experience occurred when the response matched the emotional needs of the young woman in that moment. For the majority of these experiences, the adult was a professional, such as a GP, crisis support service worker, therapist, psychologist, or teacher, as opposed to a family member.

*“… my GP was really, really good and I think it made me feel a bit more validated. He confirmed, he was like, I’ve never seen you this upset and this distressed before. And so having that external perspective on yes, you are feeling emotions that are a lot stronger than I’ve seen was really helpful.”*

(Participant 4)

*“Having someone* [peer support workers] *to talk to who had been through mental health struggles and actually understood what I was talking about was really helpful.”*

(Participant 8)

Besides the positive experience of having one’s suffering acknowledged, and a general feeling of helpfulness, validating experiences with health professionals could teach the young person that previous inappropriate responses with health professionals may not always occur.

*“… And I was like, okay, so that’s not going to be the reaction every time.”*

(Participant 10)

## 4. Discussion

The current study sought to qualitatively investigate how young women perceived adult responses to self-harm disclosure and the impact of this on the young woman. Reflexive thematic analysis of 27 semi-structured interviews with Australian young women (aged 18–24 years) endorsing past or current self-harm was conducted. The analysis generated three themes: (1) the young woman’s needs were diminished through the response; (2) the confidant’s response was not attuned to the needs of the young woman; and (3) the confidant’s response was attuned to the needs of the young woman. The diversity of responses and young women’s appraisal of these responses are consistent with previous findings [17,22]. The findings can also be mapped to three of the components of the Four Factor Model (FFM) of self-injury [38] given a young person’s propensity to engage in self-harm in response to negatively perceived responses (both automatic and social negative reinforcement) and in order to facilitate access to help and support (social positive reinforcement).

A large portion of young women described negatively perceived or unhelpful adult responses to self-harm disclosure, in particular when experiences were invalidated or dismissed. Consistent with prior literature, negative reactions and judgement after disclosing self-harm [22] included inaction, avoidance, silence, anger, dismissiveness, tearfulness, stigmatisation, or trivialisation of the self-harm [5,17,39]. Previous studies have indicated that negative responses to disclosure increase young people’s feelings of distress and self-harm behaviours [22,26]. Similarly, some young women in this study indicated that they would engage in self-harm or attempt suicide in response to negative disclosures. In alignment with Joiner’s Interpersonal Theory of Suicide (IPTS) [40], invalidating or dismissive responses to disclosure may increase a young woman’s perceived burdensomeness and decrease their sense of belonging. When this is coupled with an already increased capacity for suicide through repeated exposures to self-harm [41], it is possible that negative responses could increase suicide risk or self-harm behaviours [16,24,26]. As such, it is important that adults engaging with young people (including parents, health professionals, and educators) are provided sufficient education to respond safely to self-harm disclosures.

Disclosure may be more likely for those experiencing suicidal ideation or more severe self-harm behaviours [14]. As such, it is concerning that the findings suggest negatively perceived responses detrimentally impacted young women’s future decisions to disclose or seek help. There is contention in the literature as to whether negative experiences with disclosure influences future disclosure or help-seeking. Notably, one study demonstrated the detrimental impacts of such negative experiences on the willingness to disclose or increasing one’s caution to disclose [21], whereas another study found negative disclosure experiences was not associated with willingness to disclose again [23]. Nevertheless, the present study suggests that when young women do not receive acknowledgement and support, they perceive this to be a message that their self-harm behaviours do not require intervention and should be kept hidden. Given that disclosing self-harm can be important to ensuring that young people have access to support, it is important to ensure that the responses and support offered by adults is appropriate [33]. However, counsellors, teachers, and GPs report not feeling confident to respond to self-harm disclosures [30,41,42] and respond with higher levels of anxiety and panic [30]. The current findings suggest that emotionally modulated expressions of care and concern were well received, while expressions of anger, embarrassment, or fear were unhelpful. As such, more training may be helpful to support teachers and healthcare professionals to respond appropriately to disclosure. However, the present study found that when a healthcare professional is validating, this may teach the young woman that previous inappropriate experiences in a health setting may not always occur. Hence, it seems possible that emotionally validating experiences with disclosure may play a role in rectifying previous harmful effects of negative responses to self-harm. More research is necessary to explore this phenomenon further.

Responses which demonstrated that the adult was attuned to the young woman’s needs were perceived as helpful or meaningful in recovery from self-harm and generated insights into the importance of their own safety. Positively perceived responses to self-harm have been shown to reduce shame associated with self-harm [17], facilitate future help-seeking or disclosures, reduce future risks of suicide [17,21,25,26], and have demonstrated associations with decreased levels of depression [26]. The characteristics defining “positive” style responses vary across studies. Some include showing sympathy and emotional support, providing tangible aid [23], understanding, and acceptance [17]. However, participants in the current study preferred emotional support over the provision of tangible aid or treatment strategies. This discrepancy highlights the need for more research as being necessary to understand which responses should be prioritised by each adult and under what circumstances providing a particular support (such as providing coping strategies) may be perceived to be helpful. For instance, Ammerman and McCloskey [23] suggested that emotional support, such as listening non-judgementally to the person disclosing, may be more effective at promoting positive behavioural outcomes than if the confidant provides resources (i.e., offers to obtain medical care). However, ongoing emotional support after a disclosure is also important [22], which may be particularly important if the confidant is a parent [5,29]. Additionally, the types of responses sought may differ depending on if a condifant is a parent, healthcare provider, or another adult, but more research is necessary to specifically examine such distinctions. Nevertheless it is important that all adults that young women are engaged with are equipped with the skills to both respond appropriately to a self-harm disclosure and provide the necessary ongoing support.

### 4.1. Limitations and Future Directions

Despite critical findings, this study possesses some limitations. Firstly, the current study was part of a larger research study, and as such, the interview questions covered a broad range of experiences related to self-harm as opposed to solely exploring self-harm disclosure. As such, it was not possible to explore specific contextual factors, including interpersonal dynamics, socioeconomic status, access to mental health services, and cultural norms that may have impacted disclosure and confidant responses. Exploration of these factors in future research would be beneficial. The current interviews were conducted online, and it is possible that this may have affected the depth and nature of disclosures [43]. Age of onset of self-harm has been shown to peak in adolescence [44]; however, despite recruiting for participants aged 16–17 years, none participated in the current study. Additionally, this study did not classify what stage the young woman was at during their experience with self-harm, and the decision to disclose self-harm behaviours does not necessarily indicate readiness to cease the behaviour [16]. Similarly, some participants described instances where disclosure was unintentional or not self-directed. Processes of help-seeking are typically more effective when the choice to disclose and seek help is self-directed [16]. Hence, further research should investigate whether the perception of adult responses, and their subsequent impacts, differs depending on the young woman’s age, readiness to seek help, and circumstances of the disclosure.

Importantly, none of the young women interviewed identified as Aboriginal or Torres Strait Islander. There are cultural differences in motivations and experiences of self-harm [45], specifically amongst Indigenous populations in Australia [46]. Connectedness with community, mob, and family are all fundamental maintaining factors of Indigenous wellbeing [47,48]. Thus, the impact of adult responses to self-harm disclosure amongst Indigenous young women may be quite different to non-Indigenous young women who self-harm. Further research is needed to understand the differences across various cultural groups.

The recruitment of participants to the current study was largely through online sources. As such, the generalizability of the findings to all young women, particularly those not engaged online or comfortable discussing their self-harming behaviour, is not clear, and future research with more diverse samples is warranted. Finally, participants were not involved in checking interview transcripts or reviewing the analysis or results, and therefore, some of the interpretations made may be inaccurate. Nevertheless, the overall consensus between the reasonably large sample size provides a degree of confidence in the findings. Future research is necessary to validate these findings.

### 4.2. Implications

Despite these limitations, the current study has significant implications for future research and intervention development. The necessity of additional training and support for parents and professionals in responding to self-harm has been identified in several studies [29,30,31,32]. The current study’s findings further highlight the significance of developing resources to support adults to respond to self-harm disclosures in a way that facilitates recovery and is ultimately beneficial for the young woman, as opposed to responses that potentially cause harm. For example, there would be value in developing self-harm communication training for general practitioners to assist them in better responding to disclosures of self-harm, with an emphasis on the use of non-judgmental, non-dismissive, calm, and empathetic language. Parents and other caregivers would also benefit from interventions, such as gatekeeper training [49], that could provide them with the knowledge, skills, and confidence to better recognise and respond to self-harm in their child. Alongside these interventions is the need for evaluations to assess their effect on participant knowledge of how to respond to self-harm disclosures, comfort and confidence in managing a disclosure, and treatment engagement following disclosure. The provision of affordable and high-quality care that is accessible to young people and meets their unique needs is also needed to support safe and timely help-seeking for self-harm.

Aligned with this, the Lancet Commission on Self-Harm [50] identified a range of responses to address rising rates of self-harm. It recommends the training and supervision of health and social-care professionals in the compassionate assessment and management of self-harm, adequate service staffing, better integration of services to ensure that people who self-harm receive the help they need, and the inclusion of young people with lived experience in the co-design of interventions. The Commission recognised the challenges of addressing self-harm in low- and middle-income countries, including the criminalisation of self-harm, which acts as a substantial barrier to providing care to those who self-harm. It highlighted the need to learn from successful suicide prevention efforts by addressing upstream drivers such as social and economic conditions and means restriction. The need to support parents of young people who self-harm has been highlighted in other research [51].

## 5. Conclusions

The present study provides vital insights into how young women perceive responses to self-harm disclosure and the potential impacts of these responses. Findings suggest that dismissive (such as minimising or ignoring the self-harm) or unhelpful responses (i.e., a response that does not support meaningful change), may contribute to increased self-harm behaviours or, in the case of dismissive responses, to suicide attempts. Additionally, if an adult response elicits feelings of discomfort, shame, or invalidation, this may contribute to a young woman’s hesitation to disclose again or to engage in future help-seeking behaviour. Furthermore, adult responses that are attuned to the young woman’s needs and demonstrate care or emotional validation, such as a parent taking their daughter to the hospital, an adult acknowledging the young woman’s distress, or providing space to listen to her, can be beneficial for recovery. Whilst the present findings highlight the need to develop appropriate interventions to upskill adults in responding to self-harm disclosure, further research is needed to understand the nuances and impacts of adult responses on young women who disclose self-harm.

## Figures and Tables

**Table 1:** Three identified themes and codes.

Theme	Code	Description
The young woman’s needs were diminished through the response	Self-harm was dismissed	Responses which led to the young women feeling as though the adult was minimising, dismissing, or ignoring the self-harm disclosure.
	Young women felt uncomfortable	Responses led to the young women feeling discomfort or embarrassment or those which resulted in big emotional reactions from the confidant.
	Young women felt ashamed	Responses which made the young women feel shame or guilt for engaging in self-harm, which, while often well intentioned, this was distressing for participants.
The confidant’s response was not attuned to the needs of the young woman	Needs were misunderstood	Invalidating responses which made the young women feel as those their self-harm was misunderstood by the adult.
	Response was unhelpful	Responses which did not feel helpful despite intention to provide help. Typically experienced when engaging with healthcare professionals.
The confidant’s response was attuned to the needs of the young woman	Young women felt cared for	Responses which made the young women feel as though the adult cared for them, typically associated with an action or offer to help.
	Struggles were acknowledged	Validating and understanding responses which were attuned to the emotional needs of the young person.

## Data Availability

The data presented in this study are available on request from the corresponding author due to ethical considerations.

## Supplementary Materials

The following supporting information can be downloaded at: https://www.mdpi.com/article/10.3390/ijerph22121879/s1, Supplementary S1. Interview Guide.
